# Supplementary material for: Gene Gangs of the Chloroviruses: Conserved Clusters of Collinear Monocistronic Genes
Source: Viruses. 2018 Oct 20;10(10):576. doi: 10.3390/v10100576 (PMC6213493; doi:10.3390/v10100576)

Bin_conservation_minus_bg_ATCV1_vs_OR0704.2.2
PD−0.45874

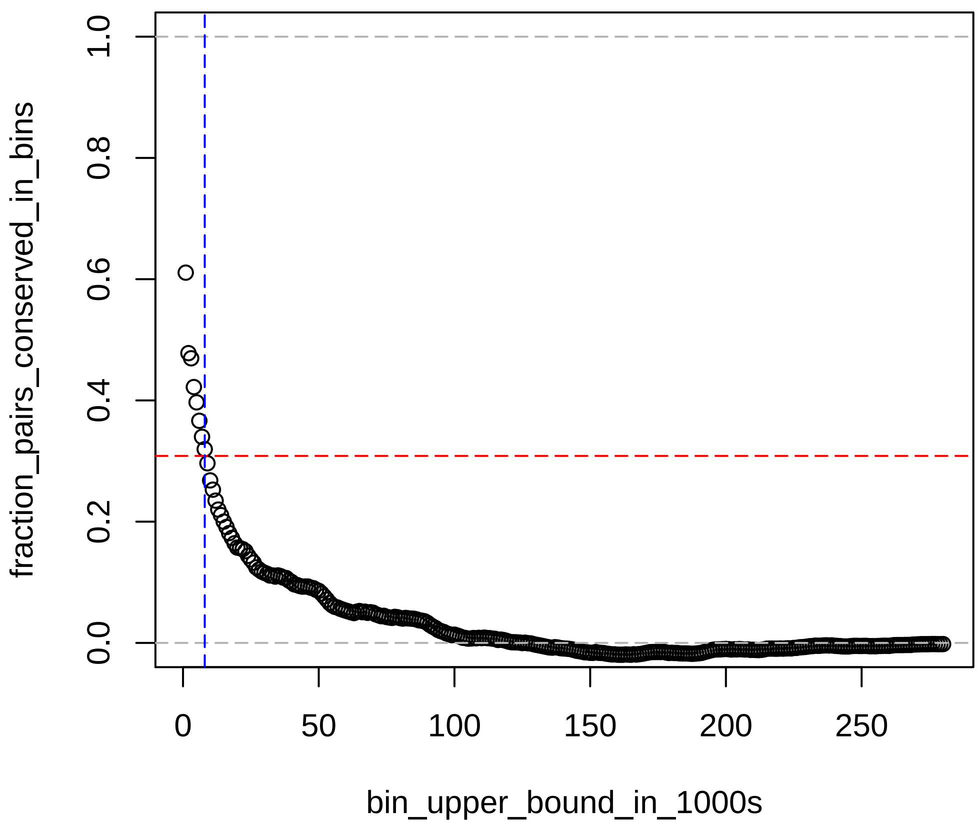

Bin_conservation_minus_bg_ATCV1_vs_OR0704.2.2
PD−0.45874

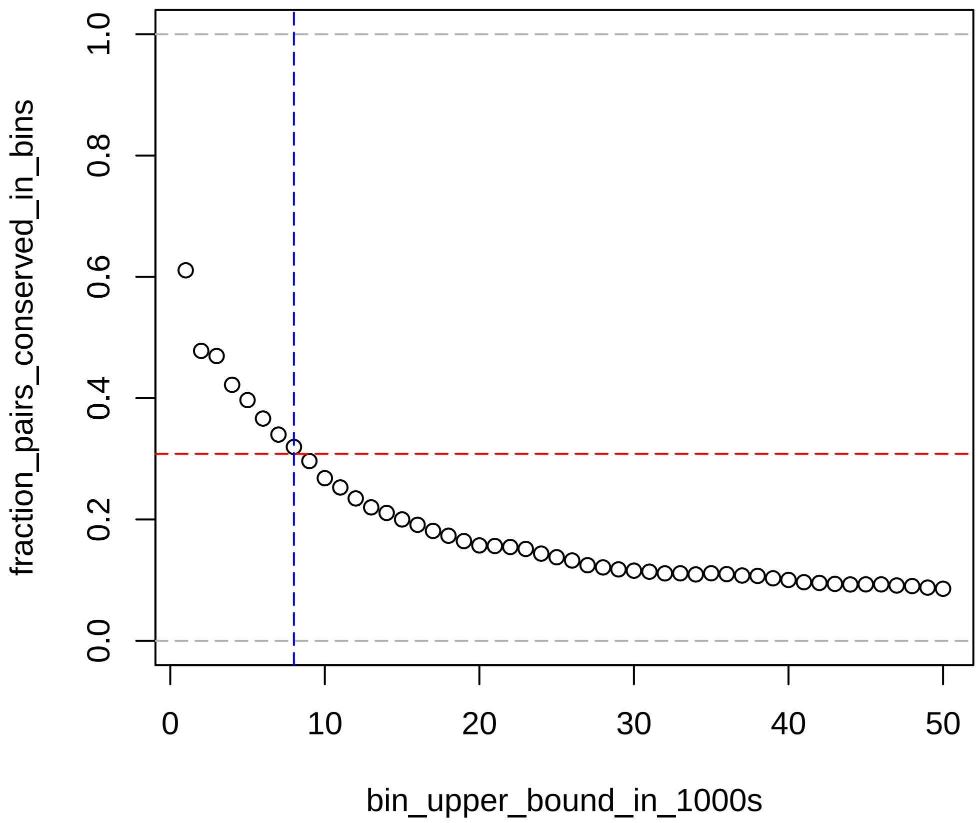

Supplement: Supplementary file 1 [file viruses-10-00576-s001.zip › viruses-363410-suppl_/supplementary/File_S7.zip!/plots_subtracted_combo/Bin_conservation_minus_background_ATCV1_vs_OR0704.2.2.pdf]
